# Incorporation of lateral microfiltration with immunoaffinity for enhancing the capture efficiency of rare cells

**DOI:** 10.1038/s41598-020-71041-7

**Published:** 2020-08-26

**Authors:** Kangfu Chen, Jacob Amontree, Jose Varillas, Jinling Zhang, Thomas J. George, Z. Hugh Fan

**Affiliations:** 1 Interdisciplinary Microsystems Group (IMG), Department of Mechanical and Aerospace Engineering, University of Florida, P.O. BOX 116250, Gainesville, FL 32611 USA; 2 J. Crayton Pruitt Family Department of Biomedical Engineering, University of Florida, P.O. Box 116131, Gainesville, FL 32611 USA; 3 Department of Medicine, University of Florida, P.O. Box 100278, Gainesville, FL 32610 USA; 4 Department of Chemistry, University of Florida, P.O. Box 117200, Gainesville, FL 32611 USA

**Keywords:** Biomedical engineering, Lab-on-a-chip, Microfluidics

## Abstract

The methods for isolating rare cells such as circulating tumor cells (CTCs) can be generally classified into two categories: those based on physical properties (e.g., size) and methods based on biological properties (e.g., immunoaffinity). CellSearch, the only FDA-approved method for the CTC-based cancer prognosis, relies on immunoaffinity interactions between CTCs and antibodies immobilized on magnetic particles. Immunoaffinity-based CTC isolation has also been employed in microfluidic devices, which show higher capture efficiency than CellSearch. We report here our investigation of combining size-based microfiltration into a microfluidic device with immunoaffinity for enhanced capture efficiency of CTCs. The device consists of four serpentine main channels, and each channel contains an array of lateral filters that create a two-dimensional flow. The main flow is through the serpentine channel, allowing the majority of the sample to pass by while the secondary flow goes through the lateral filters. The device design is optimized to make all fluid particles interact with filters. The filter sizes range from 24 to 12 µm, being slightly larger than or having similar dimension of CTCs. These filters are immobilized with antibodies specific to CTCs and thus they function as gates, allowing normal blood cells to pass by while forcing the interactions between CTCs and antibodies on the filter surfaces. The hydrodynamic force experienced by a CTC was also studied for optimal experimental conditions to ensure immunoaffinity-enabled cell capture. The device was evaluated by capturing two types of tumor cells spiked in healthy blood or a buffer, and we found that their capture efficiency was between 87.2 and 93.5%. The platform was further validated by isolating CTCs from blood samples of patients with metastatic pancreatic cancer.

## Introduction

Circulating tumor cells (CTCs) have been considered an important biomarker for early detection of cancer metastasis, therapy monitoring, and disease prognosis^1–3^. However, they are exceptionally rare, with only a few CTCs in billions of normal blood cells in each milliliter of peripheral blood^4^. As a result, their isolation is technically challenging. CellSearch is the only CTC isolation method approved by the U.S. Food and Drug Administration (FDA) for cancer prognosis and it is based on the immunological interaction between epithelial cell adhesion molecules (EpCAM) on CTCs and anti-EpCAM immobilized on magnetic particles. Similar immunoaffinity-based isolation has also been implemented in microfluidic devices to differentiate CTCs from normal blood cells^5–10^.

Over the past decade, many efforts have been made to increase CTC capture efficiency in microfluidics-enabled, immunoaffinity-based methods; some representative works are listed in Table S1 in Supplementary Information^5–7,11,12^. A prevalent and effective approach is to enhance the interaction between CTCs and the antibodies immobilized in devices. For instance, microstructures such as microposts of different sizes^13^, in various shapes^14–16^ and unique arrangements^17,18^, and on different substrate materials^5,19^ were created to change the flow patterns and increase the collisions between CTCs and capture agents on microposts. Herringbone structures were used to enhance mixing in microchannels, thus increasing the interactions between CTCs and capture agents on channel surfaces^6,7^. Nanostructures such as silicon nanopillars^11^, DNA nanospheres^20^, titanium oxide nanofibers^12^, quartz nanowires^21^, graphene oxide nanosheets^22^, and polymer nanofibers^23^ have been reported to provide more CTC-binding sites. While these methods provide more opportunities for CTCs and antibodies to interact, they do not actively prevent the cloaking effect, which refers to a CTC being loosely surrounded by platelets and other blood cells^24,25^. The cloaking effect can reduce the direct contact opportunities between the CTC and antibodies immobilized in the device.

In addition to immunoaffinity, another category of CTC isolation methods is based on cells’ physical properties such as their size^26–29^. Since CTCs are generally larger than normal blood cells, filtration using microfabricated porous membranes has been employed to isolate CTCs^30,31^.

Recently, we reported a lateral filter array microfluidic (LFAM) device that combines size-based separation with immunoaffinity-based CTC isolation to address the limitation of most platforms that use either one of these two mechanisms only^32^. Lateral filters with a size of 6–10 µm were integrated in LFAM to filter CTCs because most size-based CTC isolation devices are made up of membrane filters with a pore size around 7–8 µm^30,31^. These filters are arranged in zones from the inlet to the outlet, varying from 10 to 6 µm, with a decrease of 1 µm in each subsequent zone (Fig. S1 in the Supporting Information). As expected^33^, we found that combining the physical property (size) and biological property (immunoaffinity) of CTCs is more advantageous than using only one of these two properties as the isolation mechanism. Microfilters immobilized with antibodies gave higher CTC capture efficiency than microfilters without antibodies. Unexpectedly, however, we found an interesting phenomenon that more than 90% of captured cells are distributed in the 10-µm lateral filters, independent of the flow rate, when microfilters are immobilized with antibodies (Fig. S1)^32^. This is strikingly different from those vertical membrane filter-based CTC platforms using a pore size of 7–8 µm^30,31^. Therefore, we were wondering if our observation in 10-µm lateral filters is also true for filters larger than 10 µm.

Moreover, the integration of lateral filters with immunoaffinity could be more advantageous than conventional immunoaffinity-based CTC isolation such as herringbone-based micromixer devices^6,7^ because filters force CTCs to pass through individually, eliminating the possible cloaking effect when a CTC is surrounded by platelets and other blood cells^24,25^. The cloaking effect can reduce the direct contact opportunities between the CTC and antibodies immobilized in the device.

In this work, we report a second lateral filter array microfluidic (LFAM2) device—that contains filters ranging from 12 to 24 µm—to study the effects of combining microfilters with immunoaffinity on the enhancement in capture efficiency of CTCs. In this LFAM2 device, microfilters work more like a gate than a filter, to prevent a CTC from clumping with other cells and increase the probability of direct contacts between CTCs and antibodies immobilized on filters. The flow pattern in LFAM2 was studied and the device design was then crafted to maximize the interactions between CTCs and filters. A fluid–solid interaction (FSI) model was developed to analyze the hydrodynamic force experienced by a tumor cell to ensure active immunoaffinity-based cell capture. Different types of tumor cells were tested to evaluate the performance of the LFAM2 device. The tumor cells were spiked in healthy blood and infused into the antibody-functionalized LFAM2 device to simulate CTC isolation. Finally, LFAM2 was investigated using clinical blood samples of patients with metastatic pancreatic cancer.

## Experimental section

### Device fabrication

**Figure 1:**
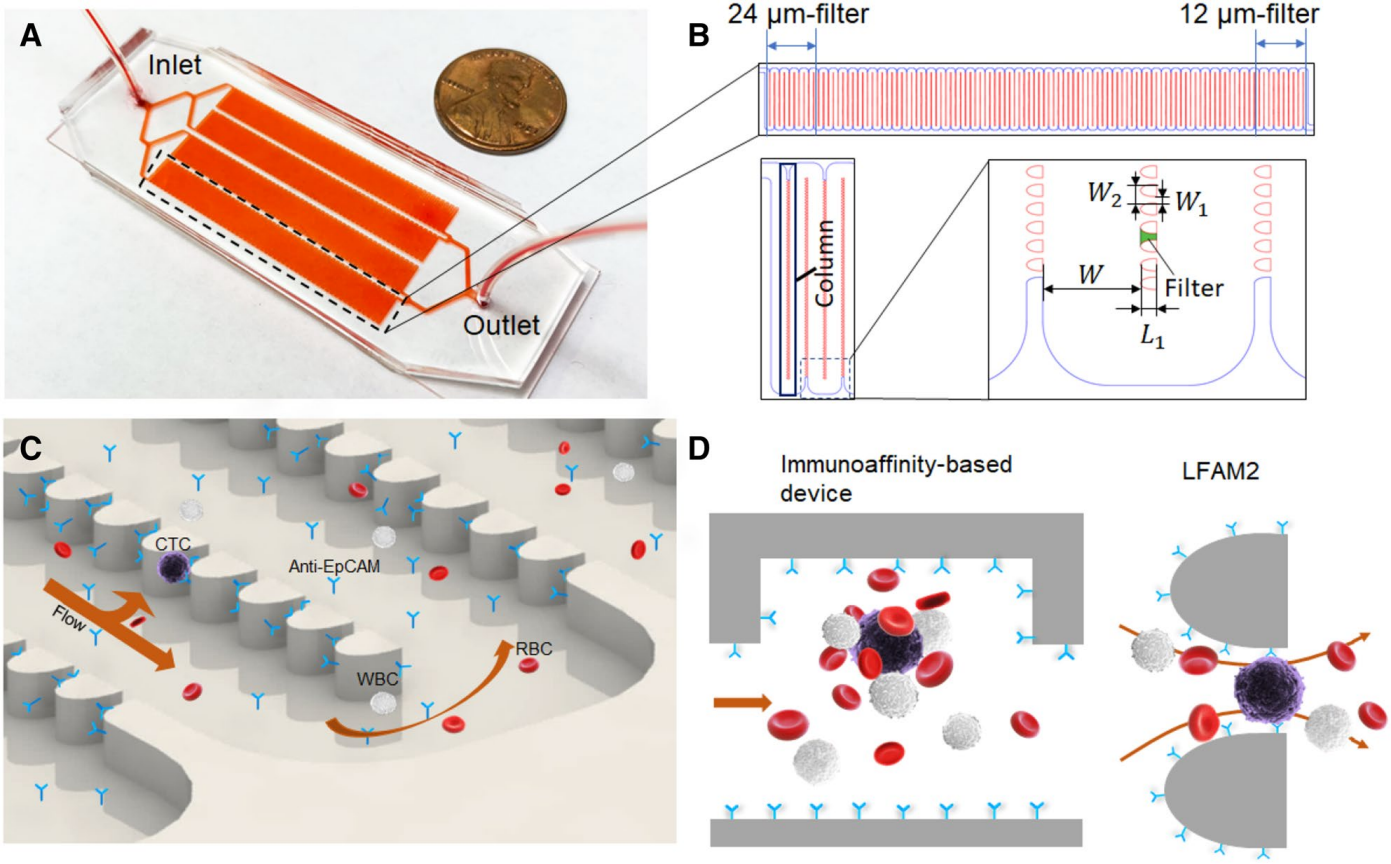
(**A**) Photo of the LFAM2 device, showing its size relative to a penny. (**B**) One serpentine main channel shown at the top, consisting of the 24-µm filter zone near the inlet and the 12-µm filter zone near the outlet. On the lower left are a “column” of filters and the “channel elbow,” which is further zoomed in on the lower right, showing the dimension marks of the serpentine main channel and filters. The device design was drawn by using AutoCAD 2016. (**C**) Illustration of a two-dimensional flow in the device, represented by one arrow along the main channel and one arrow towards lateral filters. This parking-lot-like arrangement prevents cells from clogging. Antibodies are immobilized on the surfaces of filters and channels for CTC capture. The drawing was created by using SolidWorks 2017. (**D**) Cartoons comparing a conventional immunoassay-based CTC device with LFAM2. Left: a herringbone-mixer-based CTC device using immunoaffinity capture where a CTC is cloaked by a number of blood cells. Right: a microfilter forces the direct interactions between antibodies on the filter surface and biomarkers on a CTC, while reducing the interference of other blood cells. The cartoons were drawn by using Microsoft PowerPoint.

The pattern of the LFAM2 device was sketched using AutoCAD. The CAD file was then sent out to a company (CAD/Art Services, Inc. OR) for printing on a transparency film. A dark-field film containing the pattern was taped on a 4 × 4 in^2^ glass plate to form a photomask. The pattern was transferred to a silicon wafer using photolithography while SU-8 2025 was employed as a photoresist. The silicon master was used to fabricate polydimethylsiloxane (PDMS) substrates using soft lithography. The PDMS substrate was bonded with a microscope slide after 5 min of UV ozone treatment. The device is shown in Fig. 1A and images of the filters are shown in Fig. S2.

### Cell culture

Metastatic human pancreatic cancer cells, L3.6pl, were obtained from Dr. Jose Trevino (Department of Surgery, University of Florida or UF) while human breast adenocarcinoma cells, MCF7, were provided by Dr. Carlos Rinaldi (Department of Chemical Engineering, UF), which were originally purchased from American Type Culture Collection (ATCC). Acute lymphoblastic leukemia cells CCRF-CEM were purchased from ATCC. The L3.6pl cells and MCF7 cells were cultured in DMEM media (ATCC) supplemented with 10% fetal bovine serum (FBS, GIBCO) and 100 units/mL penicillin–streptomycin (Cellgro, Manassas, VA). The CCRF-CEM cells were cultured in RPMI 1640 media (ATCC) with 10% FBS and 100 units/mL penicillin–streptomycin. All cells were cultured and maintained at 37 °C with 5% CO_2_.

### Cell sample preparation

Since L3.6pl cells and MCF7 cells are adherent cells, they were first detached by exposing them to 0.25% trypsin–EDTA for 10 min, followed by neutralization using the FBS-supplemented cell growth medium. The detached cells were then rinsed twice using Dulbecco's phosphate-buffered saline (DPBS). The resultant cells were resuspended in 1 mL of DPBS and ready for experiments. CCRF-CEM cells are suspension cells and were simply withdrawn from the flask and rinsed with DPBS twice and resuspended in 1 mL of DPBS.

Vybrant fluorescent dyes (Thermo Fisher) were used for cell labeling. The dye was added to the suspended cells at 7 µL per 10^6^ cells, followed by incubation for 20 min at 37 °C. Afterwards, the cells were rinsed with DPBS three times and resuspended in DPBS.

The initial concentration of fluorescently labeled tumor cells (in a range of 1 million cells/mL) was measured using a hemocytometer. The tumor cell solution was then diluted to desired concentrations using DPBS via tenfold serial dilutions. These cells at desired concentrations were then spiked into a DPBS buffer or healthy blood. The sample was loaded into a syringe, which was then mounted on a syringe pump and connected to the LFAM2 device through tubing. When the sample was infused into LFAM2, a magnetic bar was put in the syringe and a stirring plate was placed underneath to stir the solution, preventing cells from settling. After sample infusion, DPBS was used to wash away the waste.

### Device preparation

Microchannels in LFAM2 were primed by pumping 99% ethanol into the device, followed by washing with 300 µL of DPBS. Then, 100 µL of 1 mg/mL avidin was introduced into LFAM2 and incubated for 10 min. Avidin was physically absorbed onto the surfaces in the device. Following DPBS washing, 100 µL of 10 µg/mL biotinylated anti-EpCAM was introduced to LFAM2 and incubated for 10 min. Anti-EpCAM antibodies were immobilized in the device by avidin–biotin binding. The LFAM2 device was then washed and passivated with 300 µL of DPBS containing 1% bovine serum albumin (BSA).

### Clinical samples

All de-identified healthy blood samples were purchased from Innovative Research (Novi, MI, USA) and informed consent had been performed by the commercial source. Blood samples from patients with metastatic pancreatic cancer were obtained from the UF Health Cancer Center after the protocol was approved by the Institutional Review Board (IRB, #201501053) in accordance to national and institutional guidelines. Informed consent was obtained from all participants according to the IRB-approved protocol. The samples were collected in BD Vacutainers containing anti-coagulant sodium heparin. All samples were processed within eight hours after blood draw. A total of 2–4 mL of blood was processed by each device.

Each blood sample from a cancer patient was first mixed with equal amount of DPBS, and then added to 8 mL of Ficoll-Paque in a 50-mL centrifuge tube. The tubes were then centrifuged at 800×*g* for 30 min to separate red blood cells from nucleated cells. The buffy coat with some Ficoll-Paque and plasma were extracted out and added to a 15-mL tube. The extracted mixture was centrifuged again at 200×*g* for 10 min and the supernatant was discarded. The nucleated cells were then resuspended in 1 mL of DPBS.

The sample was infused into the anti-EpCAM functionalized LFAM2 device at 1 µL/s. After washing with DPBS at the end of cell capture, 100 µL of 4% paraformaldehyde was infused into the device and incubated for 10 min for cell fixation. After washing with 200 µL of DPBS, 100 µL of 0.2% Triton X-100 was introduced and incubated for 10 min for cell permeabilization. After washing with DPBS, a cocktail containing 60 µL of 500 nM DAPI (4′,6-diamidino-2-phenylindole), 10 µL of 10 µg/mL anti-cytokeratin (CK) labeled with fluorescein isothiocyanate (FITC), and 10 µL of 10 µg/mL anti-CD45 tagged with phycoerythrin (PE) was introduced into the device and incubated for 25 min for nuclear staining and immunocytochemistry. After washing with 500 µL of DPBS, captured cells were enumerated under a fluorescence microscope (Olympus IX71). CTCs were defined as DAPI^+^CK^+^CD45^-^, while white blood cells were DAPI^+^CK^-^CD45^+^. Triple positive cells were not considered CTCs.

## Results and discussion

### Device design

As shown in Fig. 1A, the LFAM2 device consists of four serpentine main channels, one inlet and one outlet. The layout and geometry of each main channel are given in Fig. 1B. The width of the main channel is W = 300 µm and columns of lateral filters are incorporated in the serpentine channel. The filter size is defined by the smallest width of the gap ($W_{1}$) between two obstacles. The filters are designed to be approximately ‘wedge shape’ with a wider opening in the entrance to decrease cell deformation^26^. The depth of both main channel and filters are 40 µm. The filters in the main channel are divided into 11 zones based on the filter size, with each zone consisting of 10 columns of filters and each column contains 68 filters. The filter size is 24 µm near the inlet, with each filter zone decreasing by ~ 1.1 µm. The filter size in the last zone near the outlet is 12 µm. Note that the actual filter sizes are slightly different from the design due to the printing resolution of the transparency photomask; the filters fabricated in the device were measured to be 23.8 µm and 12.3 µm, respectively (Table S2). The length of each filter is L_1_ = 50 µm. The distance between two adjacent filters is W_2_ = 58 µm.

The combination of a serpentine main channel and lateral filters is designed to produce a two-dimensional flow and prevent cells from clogging, as shown in Fig. 1C. When a cell flows through the device, it has two velocity components: one along the main channel and the other along lateral filters. Additionally, the wide main channel reduces the overall hydrodynamic resistance of the device, allowing the majority of the fluid to pass by. The parking-lot-like arrangement of filters prevent cells from clogging, which can take place in vertical membrane-based CTC filtration devices^30^.

Since the filter size is in the order of the diameter of CTCs, blood cells surrounding a CTC are dispersed to allow the uncloaked CTC to have direct interactions with antibodies on the surface of filters. This is more advantageous than typical immunoaffinity-based microfluidic devices, in which a CTC may be cloaked by platelets and other blood cells^24,25^, as illustrated in Fig. 1D.

There are significant differences between this LFAM2 device and our previous reported LFAM device^32^. The filter size in LFAM is 10–6 µm whereas that in LFAM2 is 24–12 µm. As a result, filtration plays a much larger role in LFAM due to the fact that most size-based CTC isolation devices have a membrane pore size around 7–8 µm^22,23^. In contrast, immunoaffinity plays a much larger role in LFAM2 for CTC isolation while lateral filters facilitate and enhance capture efficiency as discussed later. In addition, the filter shapes in two devices are slighter different, with one in rectangle and the other in a wedged shape. To fully understand the effect of filters on immunoaffinity capture, we first studied flow patterns using simulation.

There are also differences between LFAM2 and the device reported by Chiu’s research group^34^. Chiu’s device consists of one “main channel” for retaining large cells and two “outer channels” for collecting small cells, and the main channel is separated from two side channels by filters. Although the channels are in the serpentine shape, its primary function is to extend the channel length for efficient filtrations. In contrast, LFAM2 uses the serpentine design for more interactions between cells with antibodies on filters as discussed in the next section. Other differences include the integration of immunoaffinity in LFAM2, larger/multiple filter sizes in LFAM2 (24 µm to 12 µm) than smaller/single filter size in Chiu’s device (8 µm), and an open outlet in LFAM2 versus “a trap area at the end of the serpentine” in Chiu’s device.

### Flow pattern in LFAM2

**Figure 2:**
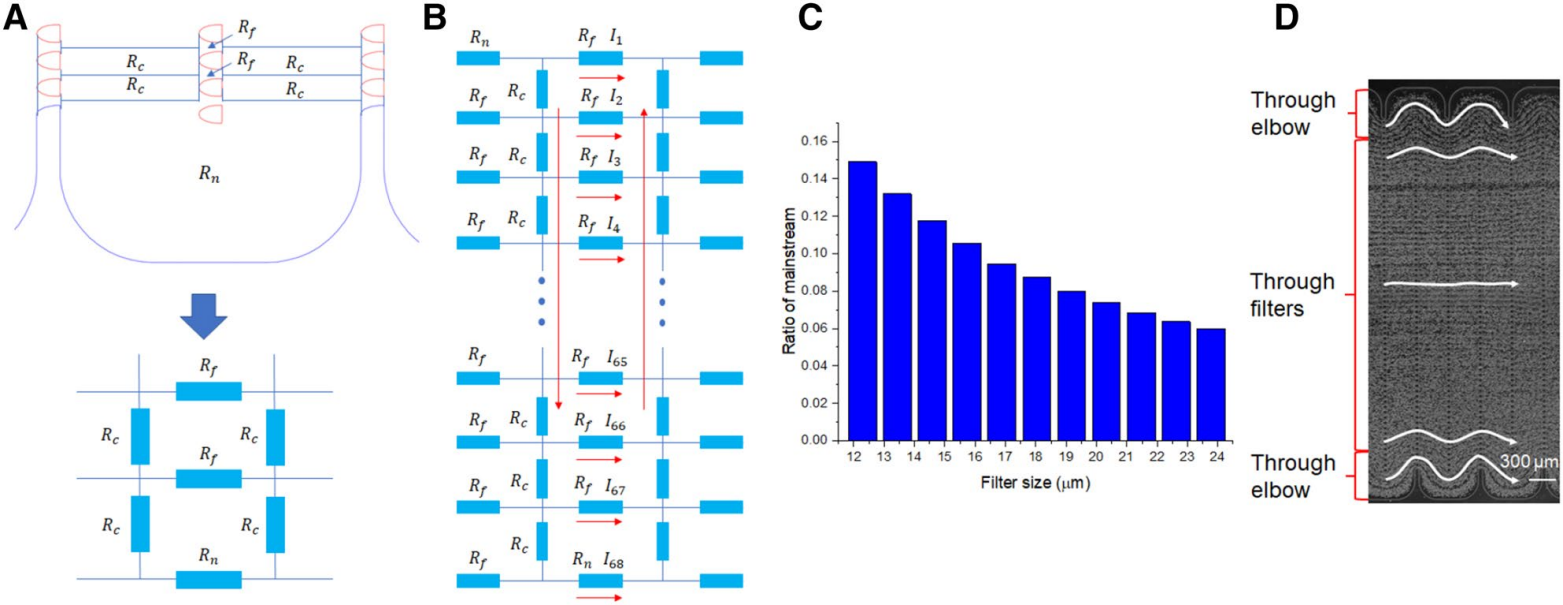
(**A**) Serpentine channel and lateral filters are modeled as a network of hydrodynamic resistors. The filters ($R_{f}$) in each column are modeled as a group of hydrodynamic resistors in parallel. The serpentine main channel is modeled as a series of hydrodynamic resistors ($R_{c}$), as well as the channel elbows ($R_{n}$). (**B**) The three basic components (*R*_*f*_, *R*_*c*_, and *R*_*n*_) are interconnected in a hydrodynamic resistor network. Their designations from 1 to 68 in the adjacent column are in a reverse order, just like the flow directions indicated by the red arrows. (**C**) The mainstream ratio calculated for all filter sizes in the LFAM2 device. (**D**) The flow pattern observed in the LFAM2 device when a blood sample is pumped through the device. The filter size in the picture is 18 µm and the scale bar is 300 µm. Five representative streaklines are added for easy visualization of the cell trajectory paths.

A theoretical model was developed to characterize the flow pattern in the LFAM2 device. The microchannel is modeled as a network of hydrodynamic resistors. As shown in Fig. 2A, the network consists of three basic components: $R_{f}$, $R_{c} ,$ and $R_{n}$. Among them, $R_{f}$ is the hydrodynamic resistance of a filter, and the serpentine main channel is considered a series of hydrodynamic resistors.

As shown in Fig. 2B, the flow rate through filter-*k* is denoted as $I_{k}$, where k is from filter 1 to 67. Since the channel elbow is in parallel with the filters, the flow rate denotation is also applicable to the channel elbow and it is denoted as $I_{68}$. Considering the total flow through the whole microchannel as $I$, using the Kirchhoff’s current law, we have:

1 $$I_{1} + I_{2} + \cdots + I_{68} = I$$

Figure 2B also shows that the subsequent columns of filters and channel elbow are in a reverse order (from the bottom to the top). The flow rates in this column are also distributed in a reverse order. The pressure drop along a certain filter $\Delta P_{k} = I_{k} R_{f}$. Using the Kirchhoff’s voltage law, we have:

2 $$\begin{array}{*{20}l} {2R_{c} (I_{68} - I_{1} ) + \left( {I_{2} - I_{1} } \right)R_{f} = 0} \hfill \\ {2R_{c} (I_{67} + I_{68} - I_{1} - I_{2} ) + \left( {I_{3} - I_{2} } \right)R_{f} = 0} \hfill \\ \cdots \hfill \\ {2R_{c} (I_{67} + I_{68} - I_{1} - I_{2} ) + \left( {I_{67} - I_{66} } \right)R_{f} = 0} \hfill \\ {2R_{c} (I_{68} - I_{1} ) + I_{68} R_{n} - I_{67} R_{f} = 0} \hfill \\ \end{array}$$

Using these equations, the flow rate in each filter and channel elbow is calculated (see the detail in Supporting Information). We call the flow rate through the channel elbow ($I_{68}$) as the mainstream flow for the subsequent column. The total flow is defined as the flow through the whole column ($I$). As a result, the mainstream ratio is defined as the ratio of flow rate in the channel elbow to the total flow rate ($I_{68} /I$). We found that the mainstream ratio largely determines the flow pattern in LFAM2 and subsequently affects the interaction frequency between cells and filters. Using this theoretical model, the flow rate distribution in the filters and the channel elbow in LFAM2 was calculated. The infusion flow rate to a serpentine main channel was set as 0.25 µL/s (to match the experiment condition at 1 µL/s in 4 main channels). For filter sizes from 12 µm to 24 µm, the mainstream ratio ranged from 6.0 to 14.9%, as given in Fig. 2C, suggesting that the majority of the flow (94.0% to 85.1%) is through filters. This theoretical result is confirmed by experimental observation shown in Fig. 2D where the flow paths go through filters except for those near the top and bottom elbows. As we discussed previously^32^, any mainstream ratio less than 50% ensures all fluid particles have opportunities to interact with lateral filters; and at 50% mainstream ratio, the flow path follows the serpentine channel with interactions with all filters in each following column.

The flow rate and flow velocity distribution in each column of filters and channel elbow for different filter sizes are given in Fig. S3. The simulated flow rate in these filters range from 0.0013 to 0.0053 µL/s, corresponding to an average velocity ranging from 2.4 to 13.2 mm/s.

### Hydrodynamic force analysis

To ensure the LFAM2 device is capable of immunoaffinity capture, we performed hydrodynamic force analysis. The hydrodynamic force experienced by a cell must be smaller than the bond force between the cell and antibodies on the filter. Our force analysis was simulated using COMSOL Multiphysics.

An incompressible flow was assumed in the microfluidic channel and the fluid flow was described by the Navier–Stokes Equation (detailed in the Supporting Information). Two dimensional (2D) fluid dynamic simulation was used to reduce computational demand^35^. The motion and deformation of a cell was achieved by a time-dependent FSI model. A linearly deformable moving mesh was created in the cell-flow interface where fluid pressure and viscous drag were imposed. The cell was considered as a solid domain with linear elasticity where solid mechanics was applied.

**Figure 3:**
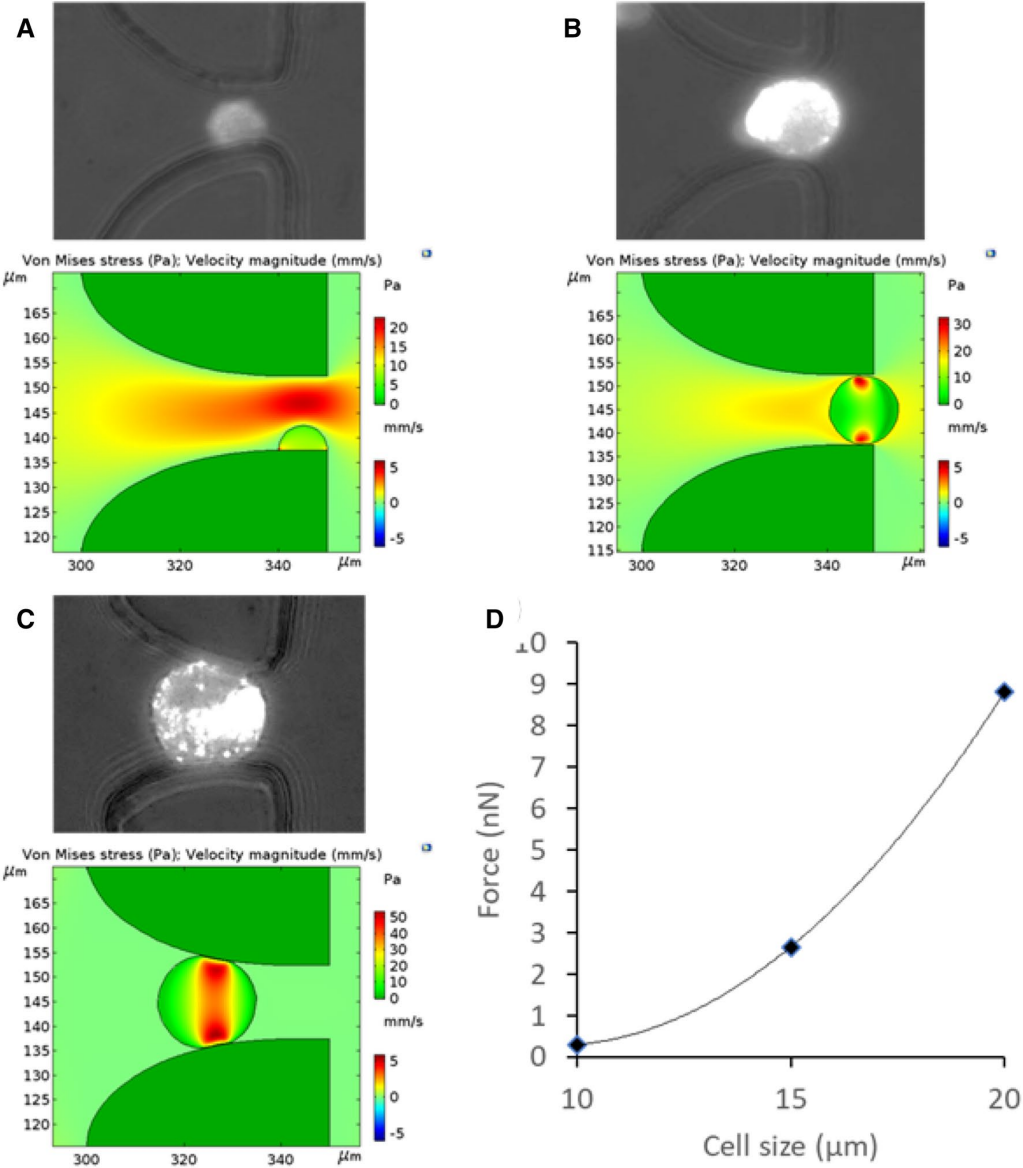
Hydrodynamic force analysis of a cell captured in a filter by immunoaffinity. (**A**) Top: picture of a cell captured in a filter by immunoaffinity. The cell has a diameter smaller than the filter. Bottom: shear stress distribution on the surface of the cell simulated using COMSOL Multiphysics. (**B**) Same as (**A**) except that the cell diameter is the same as the filter. (**C**) Same as (**A**) except that the cell diameter is bigger than the filter. (**D**) The hydrodynamic force experienced by a cell is plotted as a function of the cell size.

When a cell interacts with a filter, there are three possible scenarios: (1) the cell diameter is smaller than the filter; (2) the cell is the same size as the filter; and (3) the cell is bigger than the filter. Without considering the filtration effect (an external force added on the cell in scenario 3), the hydrodynamic force experienced by the cell must be smaller than the immunoaffinity bond force in order for the cell to be captured. We simulated the hydrodynamic force experienced by a cell using COMSOL Multiphysics, and Fig. 3 shows the results of three types of cell diameters in a 15-µm filter at a flow rate of 1.0 µL/s. The simulation results indicate that the hydrodynamic force experienced by a cell increases with the cell size. The largest hydrodynamic force experienced by a cell in all conditions simulated is 8.9 nN, which is generally smaller than the immunoaffinity bond force. For instance, the bonding force between anti-EpCAM and EpCAM on MCF7 cells is at a scale of 10^2^–10^4^ nN^36^. In other words, the bond force experienced by a cell is much bigger than the hydrodynamic force it faces, thus the LFAM2 device is capable of capturing CTCs by immunoaffinity.

Note that the hydrodynamic force experienced by a cell in LFAM2 (up to 8.9 nN) is significantly smaller than the same force in previously reported LFAM^32^ (up to 21.3 nN). Since the viability of cells captured in LFAM was 83.8 ± 5.1%^32^, LFAM2 is expected to be at least similar to, if not better than, LFAM in terms of cell viability.

### Effects of filter size and flow rate

We studied the effects of the size of lateral filters on the immunoaffinity capture efficiency in the LFAM2 device. At a given flow rate, both flow velocity and shear force are smaller in a larger filter, which is beneficial for cell capture. However, a larger filter does not effectively force the interactions between a cell and antibodies on the filter and it cannot reduce the interference of normal blood cells. To determine the best filter size for immunoaffinity capture, we evaluated the distribution pattern of cells captured in the device. L3.6pl cells and MCF7 cells were used since they are known to express EpCAM. The diameters of L3.6pl cells were 15.9 ± 3.1 µm, while that of MCF7 cells were 16.1 ± 2.5 µm^32^. About 10,000 fluorescently labeled cells were infused to the device for this study.

**Figure 4:**
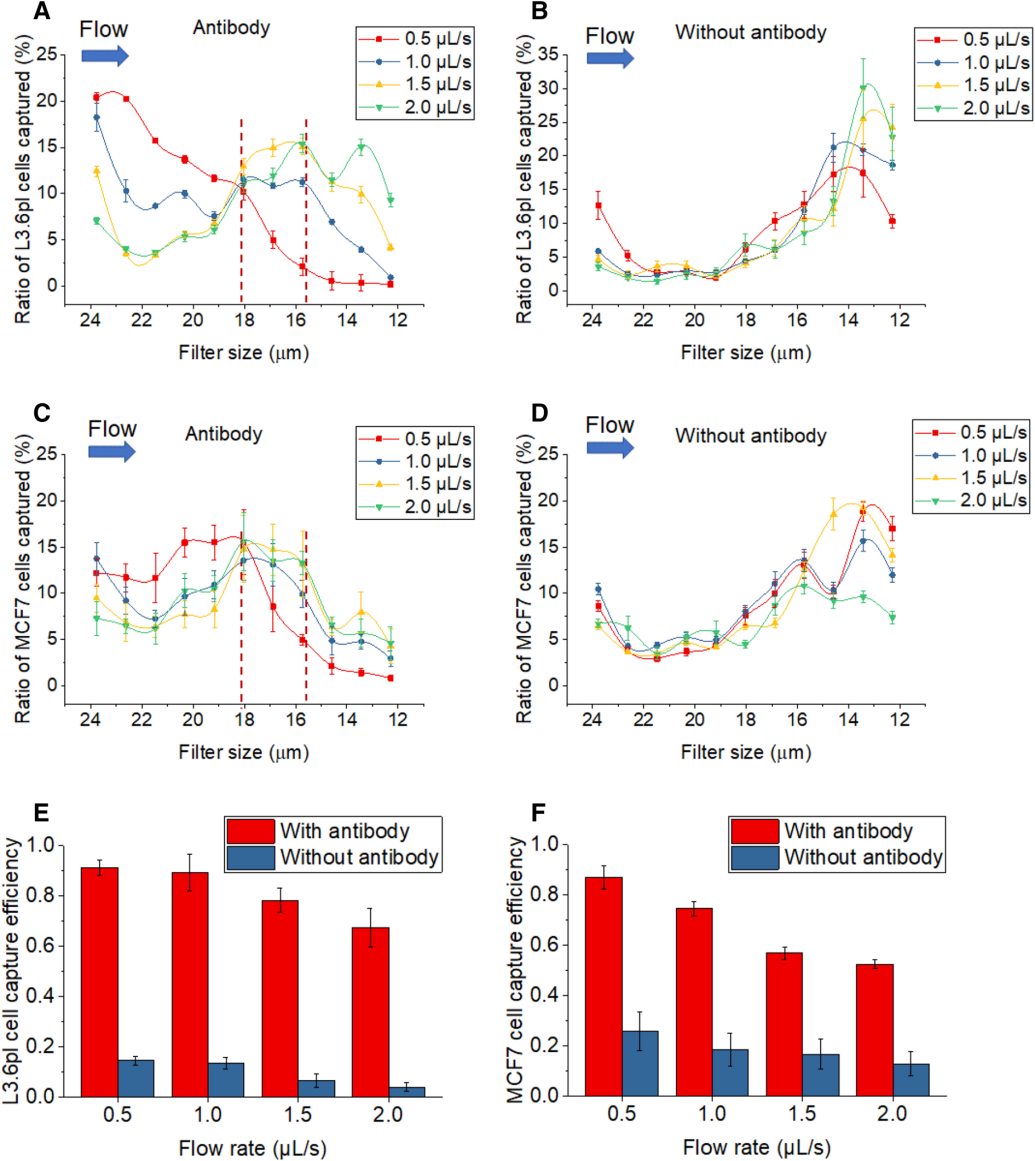
(**A**) The ratio of L3.6pl cells captured along different filter zones in the LFAM2 device immobilized with antibodies, under different flow rates. The vertical dotted lines indicate the filter zones with high cell capture. (**B**) Same as A except in the device without antibody immobilized. (**C**) Same as A except MCF7 cells were used. (**D**) Same as (**C**) except in the device without antibody immobilized. (**E**) L3.6pl cell capture efficiency in the LFAM2 device as a function of the flow rate. (**F**) Same as (**E**) except for MCF7 cells.

We studied the cell capture locations when the tumor cells were pumped into the device, from 24-µm filters near the inlet to 12-µm filters near the outlet. We evaluated the results using a parameter called capture ratio, which was defined as the ratio of the number of cells captured in a specific filter zone to the total number of cells captured in all zones. Figure 4A,C show the cell distribution pattern of L3.6pl cells and MCF7 cells in anti-EpCAM-functionalized LFAM2 devices under different flow rates. Capture ratio in the front half of the microchannel (24-µm-filter zone to 18-µm-filter zone) decreases with increasing flow rates for both cell lines, because a higher flow rate produces a higher shear force that makes tumor cells more difficult to be captured by immunoaffinity^37^. Capture ratio in the front half for L3.6pl cells is generally higher than that for MCF7 cells, likely because L3.6pl cells express more EpCAM than MCF7 cells (thus easier to be captured by anti-EpCAM on filters)^7,38^. In contrast, the capture ratio in the back half of the microchannel (18-µm-filter zone to 12-µm-filter zone) increases with the flow rate, with a plateau of the capture ratio in the region between the 18-µm-filter and 15.7-µm-filter zones except for a flow rate at 0.5 µL/s. Note that the filter size in these zones are between 113% and 99% of the diameters of the two types of tumor cells, suggesting that filters with a size slightly larger than or similar to the cell diameter give the highest enhancement effect to immunoaffinity-based cell capture. For filters significantly larger than the cell diameter, the enhancement effect to immunocapture is reduced; for filters significantly smaller than the cell diameter, the enhancement effect is less relevant because the filtration mechanism starts to dominate.

As a comparison, we also studied the cell distribution pattern in the LFAM2 device without anti-EpCAM. As expected (Fig. 4B,D), cell capture primarily took place between the 15.7-µm-filter and 12-µm-filter zones where the filter size is smaller than the cell diameter, for all flow rates, because filtration is the only isolation mechanism.

We also studied the capture efficiency in these devices as shown in Fig. 4E,F. Capture efficiency is defined as the number of tumor cells captured in the device to the total number of tumor cells spiked in the sample. Without anti-EpCAM, the device has a low CTC capture efficiency. This is expected because the filter sizes in LFAM2 are much bigger than 7–8 µm, the membrane pore size in the vertical filters-based CTC devices. It’s not difficult for a L3.6pl or MCF7 cell to deform by ~ 23% to pass the 12-µm filter^26^. However, when the LFAM2 device was functionalized with anti-EpCAM, the capture efficiency increased dramatically. For L3.6pl cells, the capture efficiency was (91.3 ± 3.0)%, and for MCF7 cells, the capture efficiency was (87.2 ± 4.7)%, both at a flow rate of 0.5 μL/s. These results also indicate that immunoaffinity plays a much bigger role in the antibody-functionalized LFAM2 device. As expected, LFAM2 shows slightly lower capture efficiency than previously published LFAM^32^ due to the larger filter sizes in LFAM2 even though the difference between two devices is statistically not significant at a low flow rate (0.5, 1.0, and 1.5 µL/s) as shown in Fig. S4.

Note that the location of filters has an effect on their cell capture efficiency. If the size of all filters is the same or very similar, the filters near the inlet interact with cells first, thus capturing more cells than those filters near the outlet. If the filter size near the inlet is significantly larger than the filters near the outlet, the location of filters will play a role with the filter sizes and their size ratios relative to tumor cells.

### Tumor cell isolation

**Figure 5:**
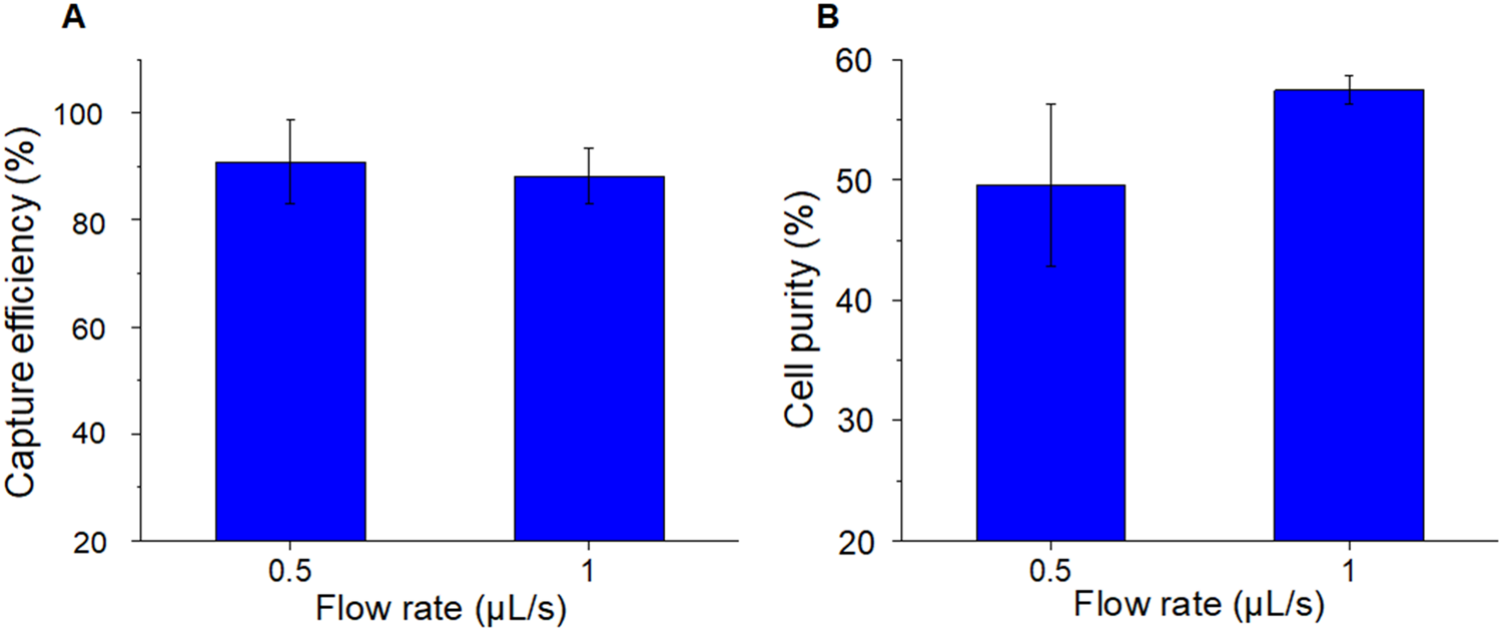
Tumor cell isolation from a mixture of L3.6pl cells (target cells) and CCRF-CEM cells (control cells). (**A**) Capture efficiency of L3.6pl cells in the antibody-functionalized LFAM2 device at a flow rate of 0.5 µL/s and 1 µL/s. (**B**) Cell purity of the same experiments in (**A**).

In addition to the capture efficiency (sensitivity) discussed above, we also studied the specificity of LFAM2 for tumor cell isolation by mixing 1,000 L3.6p1 cells (target cells) with 20,000 CCRF-CEM cells (control cells, which do not express EpCAM). The specificity is represented by a term called cell purity, which is defined as the number of target cells captured over the number of total cells captured (both target and control cells). When the cell mixture was infused into the LFAM2 device, the capture efficiency of target cells was (90.8 ± 7.8)% at a flow rate of 0.5 µL/s, and (88.2 ± 5.2)% at 1.0 µL/s (Fig. 5A), which are comparable with the data in Fig. 4E (when only target cells were used). For the same experiments, cell purity was (49.5 ± 6.7)% and (57.5 ± 1.2)% at a flow rate of 0.5 µL/s and 1.0 µL/s, respectively (Fig. 5B). The cell purity data is comparable to those in the literature (Table S1), though experimental conditions among these efforts are not exactly the same. Since the ratio of the target cells to the control cells is 1:20, the cell purity can be used to calculate that about ~ 97% control cells were removed at a flow rate of 1.0 µL/s. Note that we didn’t observe more clogging in smaller filters than in larger filters probably because the flow velocity increased in smaller filters and the nearby main channel due to the principle of mass conservation.

**Figure 6:**
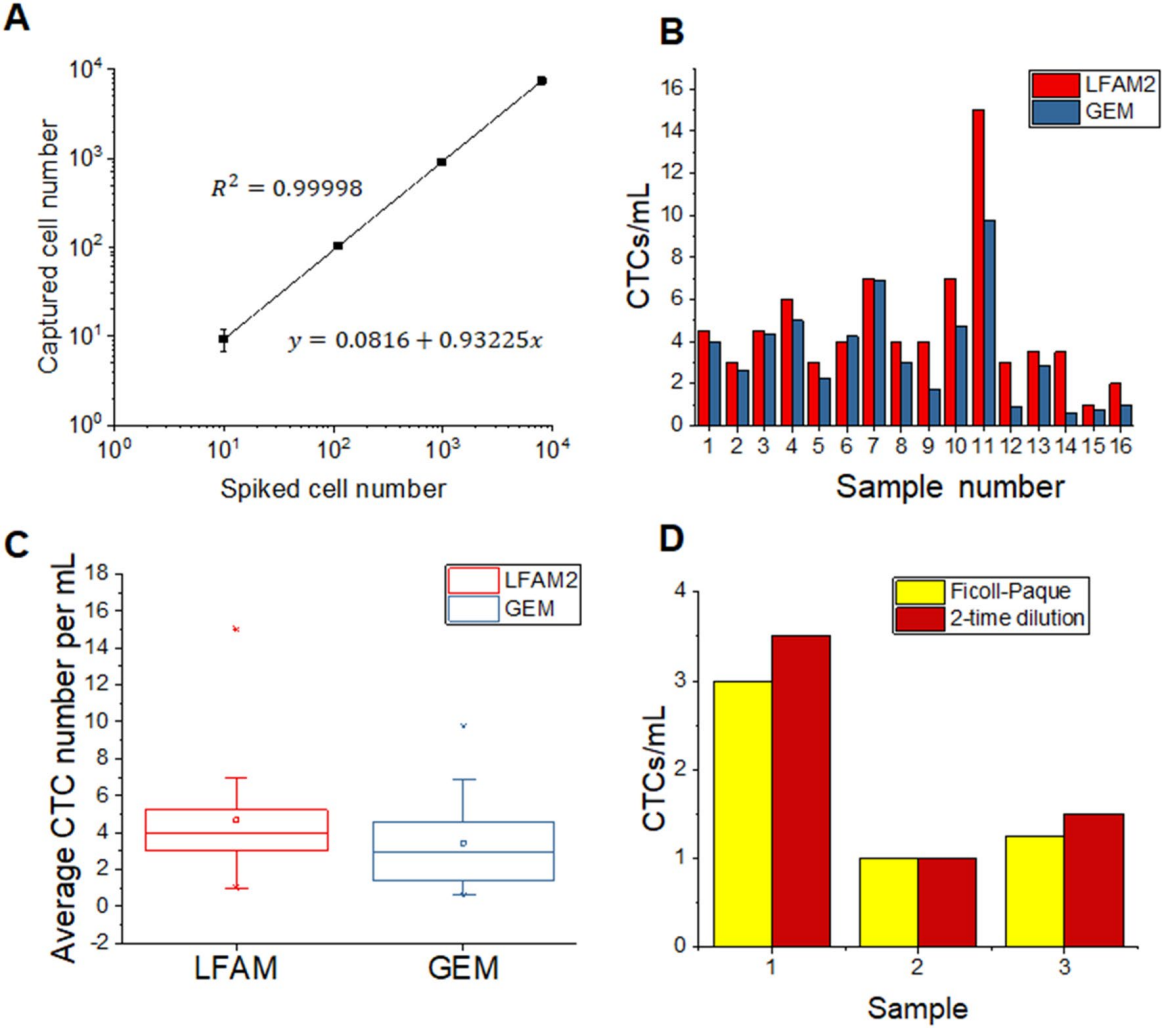
(**A**) Calibration curve between the number of L3.6pl cells captured in the LFAM2 device and the number of cells spiked in healthy blood. (**B**) CTCs per mL of blood samples from cancer patients, enumerated using either LFAM2 or GEM. (**C**) Box plots of the results in (**B**) with the average and range of CTCs per mL. (**D**) Comparison between two blood preparation methods, Ficoll-Paque and 2-time diluted whole blood, prior to infusion into LFAM2.

To mimic CTC capture in the clinical condition, 10 to 10,000 L3.6pl cells were spiked to 1 mL of 2-time diluted blood and then infused into the anti-EpCAM functionalized LFAM2 device at 1.0 µL/s. The calibration curve between the number of L3.6pl captured and the number of cells spiked is shown in Fig. 6A. The captured-cell/spiked-cell ratio maintains good linearity with a slope close to 1 for a range of three orders of magnitude in the cell concentration. The capture efficiency was as high as (93.5 ± 0.5)%. The similar spiking tests of 50 to 50,000 L3.6pl cells using a previously reported, geometric enhanced micromixer (GEM) device^7^ gave capture efficiency between 89 and 92%.

### CTC isolation from clinical samples

We evaluated the LFAM2 device for CTC isolation using blood samples of patients with metastatic pancreatic cancer. Pancreatic cancer patients are known to be low in the CTC number, thus their blood samples can be used to show the sensitivity of LFAM2. These clinical samples were also used to compare LFAM2 with a previously reported GEM device^7^. Clinical samples of 2–4 mL were prepared with Ficoll-Paque to remove plasma and red blood cells. The extracted mononucleated cells (primarily white blood cells and CTCs) were resuspended in 1 mL of DPBS. The sample was then processed for CTC isolation using either LFAM2 or GEM, both of which were immobilized with anti-EpCAM. The same clinical sample was processed in both devices for performance comparison. We only considered DAPI^+^/CK^+^/CD45^−^ cells as CTCs. CTCs were detected in all 16 clinical samples using LFAM2, ranging from 1 to 15 CTCs/mL as shown in Fig. 6B. Similarly, GEM also detected CTCs in all samples, but ranging from 1 to 10 CTCs/mL. Their box plots with the CTC range and average are given in Fig. 6C; the average CTC number detected in the LFAM2 device was 4.7 CTCs/mL, while it was 3.4 CTCs/mL in GEM devices, indicating that LFAM2 (with a combination of filtration and immunoaffinity) has higher capture efficiency than GEM (with immunoaffinity only) in general. However, there is no statistically significant difference (p < 0.05) between LFAM2 and GEM because of the large CTC variations among heterogeneous patient population.

We also compared two sample preparation methods, (1) using Ficoll-Paque in this work and in the literature^39,40^, and (2) using whole blood with 2-time dilution^32,41^. As explained before, the 1:1 dilution of a blood sample with a DPBS buffer is to reduce the effects of viscosity variation among different patients on the flow characteristics in LFAM2^30,40^ Fig. 6D shows the comparison between the two preparation methods used for CTC enumeration. The data suggests that using 2-time diluted whole blood gives higher CTC enumeration. This result is in agreement with ~ 78% recovery rate of cells after the Ficoll-Paque protocol^42^, in which loss of target cells is possible. More importantly, LFAM2 is amenable to CTC isolation using whole blood after 2-time dilution.

In addition, we studied the size of CTCs captured in LFAM2; the result is shown in Fig. S6. Since most CTCs are not strictly round^42^, their major and minor axes were measured. The average major axis of CTCs was measured 14.8 ± 5.4 µm and the minor axis was 10.9 ± 3.5 µm.

## Conclusion

A lateral filter array microfluidic (LFAM2) device is developed in this work for highly efficient isolation of CTCs. The device consists of serpentine main channels and lateral filters, which are designed to induce a 2D flow and prevent cells from clogging. The lateral filters also force direct interactions between target cells and antibodies immobilized on the filter surfaces while reducing the interference of other cells. We found that when the filter size is slightly bigger than or close to the diameter of target cells, the enhancement effect of filters on immunoaffinity capture is optimal. The antibody-functionalized LFAM2 device gave excellent capture efficiency and good cell purity when tumor cells in artificial samples were evaluated. When it was applied for CTC isolation from blood samples of patients with metastatic pancreatic cancer, LFAM2 showed better capture efficiency than the previously reported GEM that is based on immunoaffinity capture and micromixers.

We would like to point out two key differences between LFAM2 and those microfluidic devices containing pillar arrays for CTC isolation^5,19^. (1) In LFAM2 (as shown in Fig. 1C), one fluid flow is in the filter direction and the other is perpendicular to the filter flow. In those pillar devices, however, all flows are in the pillar direction. The benefits of two directional flows in LFAM2 include less clogging. (2) More importantly, the distance between pillars is much larger than the filter size in LFAM2, resulting in different interaction mechanisms in two types of devices. In the pioneering paper by Toner’s group^5^, the distance between pillars is 50 µm, and the interactions between cells and the capture agents on pillars are forced by shifting pillars 50 µm after every 3 rows of pillars, with distorting flow streamlines. In contrast, LFAM2 forces the direct interactions between cells and the capture agents on filters due to a much smaller filter size.

It is well known that CTCs are heterogeneous, and some CTCs may lose EpCAM due to epithelial-to-mesenchymal transition. This challenge can be addressed by applying an antibody against a different marker (e.g., CD133 associated with cancer stem cells^43^) or a panel of antibodies rather than anti-EpCAM alone. For our LFAM2 device, filters increase the interaction opportunities between CTCs and antibodies immobilized. Note that filters are not biased against any antibody, thus a LFAM2 device functionalized with a panel of antibodies can address the CTC’s heterogeneity in protein expression. Moreover, a range of filter sizes designed in LFAM2 also help address the CTC’s heterogeneity in the size, creating an excellent platform. In addition, LFAM2 may be useful for the isolation of CTC clusters through the combined filtration and immunoaffinity mechanisms, though the filter size may need some adjustment. CTC clusters are generally larger than single CTCs, thus they are easier to be captured by filters. And as long as one cell in a CTC cluster expresses a biomarker of interest, the cluster can be captured by the corresponding antibodies on the surfaces of the filters.

## Supplementary information

Supplementary Information
