# Supplementary material for: Incorporation of lateral microfiltration with immunoaffinity for enhancing the capture efficiency of rare cells
Source: Sci Rep. 2020 Aug 26;10:14210. doi: 10.1038/s41598-020-71041-7 (PMC7450051; doi:10.1038/s41598-020-71041-7)
Supplement: Supplementary file 1 — Supplementary Information [file 41598_2020_71041_MOESM1_ESM.pdf]

Supporting Information

Incorporation of Lateral Microfiltration with Immunoaffinity for Enhancing the Capture Efficiency of Rare Cells

Kangfu Chen[a], Jacob Amontree[a], Jose Varillas[b], Jinling Zhang[a], Thomas J. George[c] and Z. Hugh Fan[a,b,d,*]

[a] Interdisciplinary Microsystems Group (IMG), Department of Mechanical and Aerospace Engineering, University of Florida, P.O. BOX 116250, Gainesville, FL, 32611, United States
[b] J. Crayton Pruitt Family Department of Biomedical Engineering, University of Florida, P.O. Box 116131, Gainesville, FL, 32611, United States
[c] Department of Medicine, University of Florida, PO Box 100277, Gainesville, FL, 32610, United States
[d] Department of Chemistry, University of Florida, P.O. Box 117200, Gainesville, Florida 32611, United States

*Author to whom the correspondence should be addressed. Email: hfan@ufl.edu.

Comparison of representative CTC isolation methods and LFAM2

Several representative CTC isolation methods are listed in **Table S1,** and briefly explained as follows. The "CTC-Chip" contains microposts to increase the interactions between CTCs and antibodies immobilized in the microfluidic device.[1] Both "GEM" and "HB" chips consist of herringbone structures to produce mixing effects in the microfluidic devices to increase the interactions between CTCs and antibodies.[2,3] "SiNP" and "TiO_2" devices used either silicon nanopillars (SiNP) or electrospun TiO_2 nanofibers to increase the surface area for more antibodies to be immobilized in the device.[4,5] While these conventional devices provide more interaction opportunities between CTCs and antibodies, they cannot prevent the cloaking effect, which refers to a single CTC being surround by platelets and other blood cells. To address the issue, the LFAM2 device in this work combines filtration with immunoaffinity, and the lateral filters in the device force each cell to flow through them individually. As a result, CTCs have more chances to be captured by antibodies immobilized on the surface of filters.

Table S1. Comparison among representative CTC isolation methods.

Devices	Capture efficiency	Cell Purity	Throughput	Samples	References
CTC-Chip	60-80%	~50%	1-2 mL/h	Whole blood or Lysed blood	1
GEM chip	88%	~70%	3.6 mL/h	Lysed blood	2
HB chip	~90%	>50%	1.2 mL/h	Whole blood	3
SiNP	45~65%	NA	~1 mL/h	Whole blood	4
TiO_2	>45%	NA	1 mL/h	Whole blood	5
LFAM2	>90%	50-60%	3.6 mL/h	2-time diluted blood or Ficoll Paque treated	this work

Table S2. Designed filter size and corresponding values referred to in the manuscript.

Designed filter size (μm)	Measured filter size (μm)	Size referred to in the main text (μm)
10	12.3	12
11	13.4	-
12	14.6	-
13	15.7	15.7
14	16.9	-
15	18.0	18.0
16	19.2	-
17	20.3	-
18	21.5	-
19	22.6	-
20	23.8	24

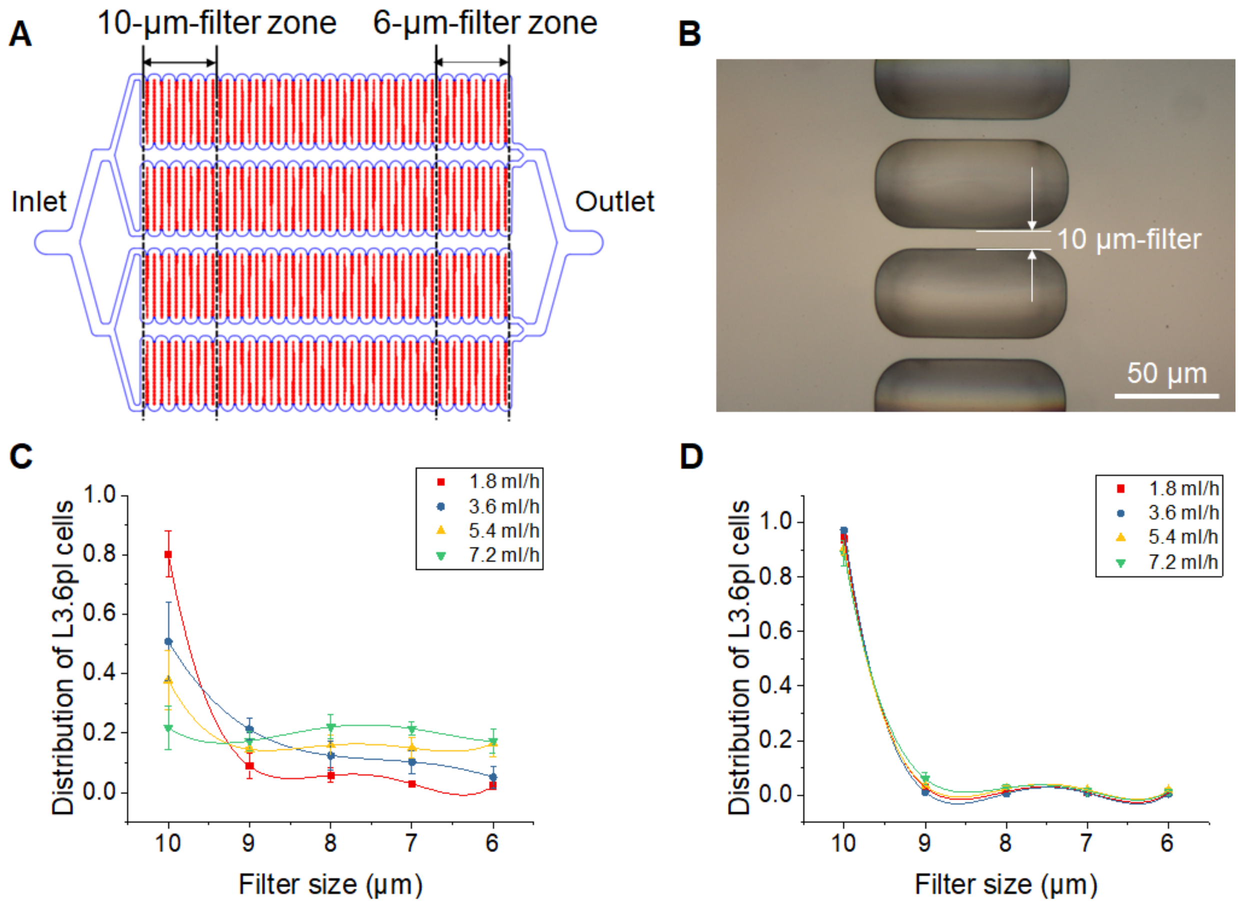

Figure S1. (A) The layout of the previously reported device.[6] It consists of four serpentine main channels incorporated with arrays of lateral filters. The filter size ranges from 10 μm near the inlet to 6 μm near the outlet. (B) Picture showing the shape of lateral filters. (C) Distribution pattern of tumor cells captured without antibodies immobilized, showing that more cells are captured downstream (i.e., 9 μm to 6 μm filter zones) with increasing flow rate. (D) Same as (c) except for the device with antibody immobilized, indicating over 90% of immunoaffinity-captured cells are in the 10-μm filter zone, independent of the flow rate.[6]

LFAM2 Device Fabrication

Fabrication of the LFAM2 device includes two steps: a silicon master using photolithography, and PDMS substrates using soft lithography.

The silicon master was fabricated from the pattern designed on the photomask. A silicon wafer was first soaked in 99+% acetone for 10 minutes to remove organic impurities. After washing with isopropyl alcohol and deionized (DI) water, the silicon wafer was soaked in Piranha solution for 5 minutes to remove any organic or inorganic impurities. After washing with large amount of running DI water, the silicon wafer was treated with buffered oxide etchant (BOE) for 30 seconds to remove silicon oxide. The wafer was washed with DI water again and dried in an oven at 120 °C for 10 minutes. After treating with hexamethyldisilazane (HMDS), the silicon

wafer was spin-coated a layer of SU8 2025 photoresist. The thickness of the photoresist was about 40 μm, controlled by spinning speed. The SU8-coated silicon wafer was then put on a hotplate for soft bake. The temperature was increased from room temperature (20°C) to 85°C at a heating rate of 120°C/hour and maintained at 85°C for 90 minutes. The dried silicon wafer was directly contacted with the photomask and exposed under UV light. The exposure dose was chosen under manufacturer's instruction. The exposed SU8 photoresist polymerized and the pattern from the photomask was transferred to the SU8 photoresist. After the silicon wafer was heated on the hotplate at 95°C for 10 minutes, it was developed for 8 minutes using SU8 developer to remove un-exposed photoresist. The silicon wafer was then put in the oven for hard bake at 120°C for 20 minutes, and thermal cracks in the SU8 photoresist disappeared after hard bake. The actual thickness of the SU8 microstructures on the silicon master was measured using Dektak 150 surface profiler (Veeco, NY).

Using the silicon master, a PDMS substrate was fabricated as follows. An aluminum foil bowl was made to hold the silicon master in the bottom. Fully mixed liquid PDMS prepolymer (base/curing agent = 10:1) was placed on the silicon master. The prepolymer-loaded foil bowl was put in a vacuum chamber to remove bubbles from the mixture. The bowl was then cured in an over at 65°C for at least 4 hours. After polymerization, a transparent elastic substrate was formed. The PDMS was then peeled off from the silicon master, edges were trimmed to fit a microscope slide and holes were punched to form the inlet and outlet. The PDMS substrate and a glass microscope slide were treated with UV ozone for 5 minutes and bonded together to form the final LFAM2 device. **Figure S2** shows images of the PDMS substrate using a scanning electron microscope.

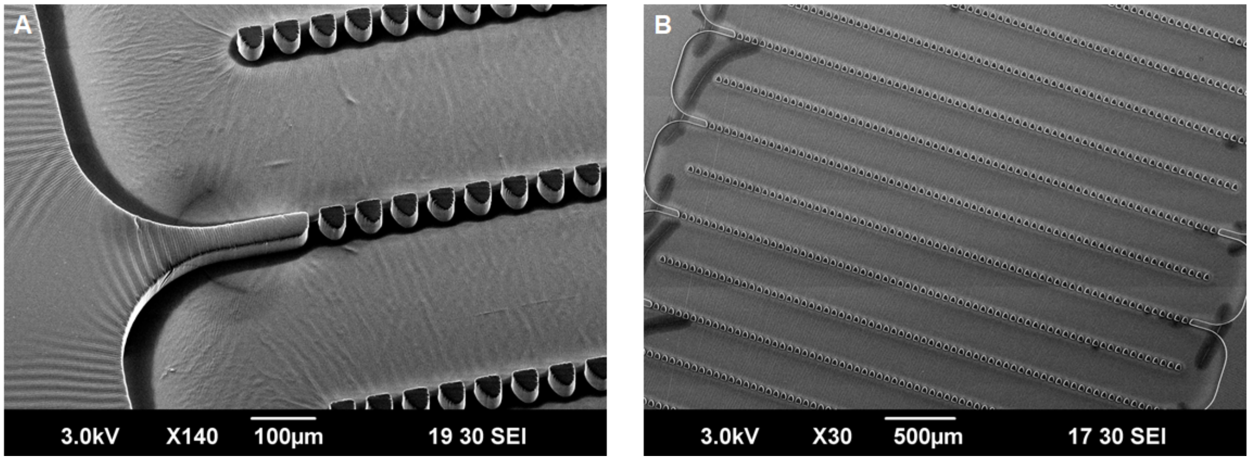

Figure S2. Scanning electron micrographs of the LFAM2 device showing the serpentine main channel and the arrangement of lateral filters.

Flow Pattern in LFAM2

A theoretical model was developed to study the flow pattern in the microfluidic device. For a laminar flow, pressure drop ΔP is proportional to flow rate Q using Stokes Law. In microfluidics, a microchannel can be modeled as a hydrodynamic resistor R_h, wherein $\Delta P = R_h Q$. The hydrodynamic resistors network is analogous to an electrical circuit, wherein the Kirchhoff's Laws are applicable.

The Kirchhoff's Current Law (KCL) and Kirchhoff's Voltage Law (KVL) given in the main text can be further arranged in a matrix form

$$\begin{bmatrix} -2R_c-R_f & R_f & & & & & & & & 2R_c \\ -2R_c & -2R_c-R_f & R_f & & & & & & 2R_c & 2R_c \\ -2R_c & -2R_c & -2R_c-R_f & R_f & & & & 2R_c & 2R_c & 2R_c \\ -2R_c & -2R_c & -2R_c & -2R_c-R_f & R_f & & 2R_c & 2R_c & 2R_c & 2R_c \\ & & & & \vdots & & & & & \\ -2R_c & -2R_c & & & & & & -R_f & 2R_c+R_f & 2R_c \\ -2R_c & & & & & & & & -R_f & 2R_c+R_n \\ 1 & 1 & 1 & 1 & 1 & & & & 1 & 1 \end{bmatrix} \begin{Bmatrix} I_1 \\ I_2 \\ I_3 \\ I_4 \\ \vdots \\ I_{66} \\ I_{67} \\ I_{68} \end{Bmatrix} = \begin{Bmatrix} 0 \\ 0 \\ \\ \\ \vdots \\ 0 \\ 0 \\ I \end{Bmatrix}$$

Using the matrix, the flow rate and average flow velocity in each filter and the channel elbow can be calculated. **Figure S3** gives the flow rate and flow velocity distribution in each column of filters with different filter sizes.

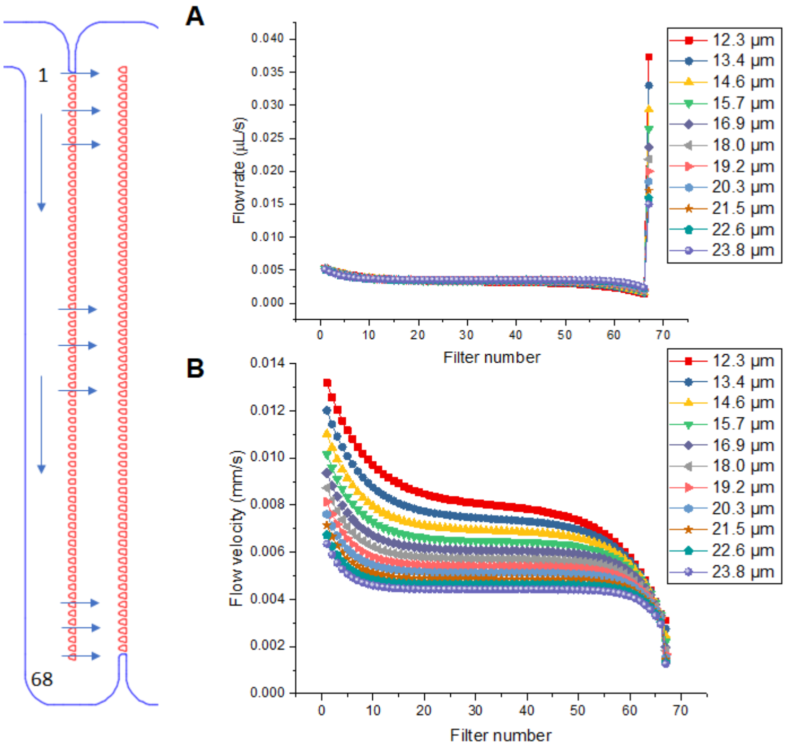

Figure S3. (A) Flow rate distribution in each column of different filter size as compared with flow rate in the channel elbow (#68). (B) Flow velocity distribution in each column of different filter size as compared with flow velocity in the channel elbow.

Simulation of hydrodynamic force

To ensure the LFAM2 device for immunoaffinity-based cell capture, hydrodynamic force analysis was carried out. Hydrodynamic force analysis for captured cells by the filters were

simulated using a Fluid-Structure Interaction (FSI) model in COMSOL Multiphysics. The fluid flow is expressed by Navier-Stokes equation:

$$\rho\nabla \cdot \boldsymbol{V} = 0 \tag{7}$$

$$\frac{\partial \boldsymbol{V}}{\partial t} + \rho(\boldsymbol{V} \cdot \nabla)\boldsymbol{V} = \nabla \cdot [-p + \mu(\nabla \boldsymbol{V} + (\nabla \boldsymbol{V})^T)] + \boldsymbol{F} \tag{8}$$

where ρ is the fluid density; $\boldsymbol{V}$ is flow velocity; t is time; μ is dynamic viscosity; $\boldsymbol{F}$ is the external force; p is the pressure. On the fluid-structure interface, the governing equations are given as

$$\boldsymbol{V} = \boldsymbol{V_w} \tag{9}$$

$$\boldsymbol{V_w} = \frac{\partial \boldsymbol{U_{solid}}}{\partial t} \tag{10}$$

$$\boldsymbol{\sigma} \cdot \boldsymbol{n} = \nabla \cdot [-p + \mu(\nabla \boldsymbol{V} + (\nabla \boldsymbol{V})^T)] \cdot \boldsymbol{n} \tag{11}$$

where $\boldsymbol{V_w}$ is the velocity of the moving cell. $\boldsymbol{U_{solid}}$ is the displacement of the cell. $\boldsymbol{\sigma}$ is the stress on the cell.

Comparison between LFAM and LFAM2

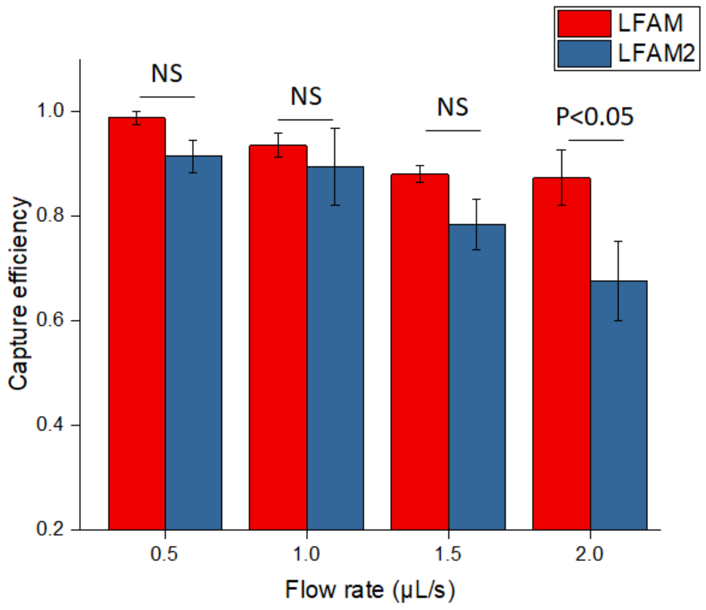

Figure S4. Comparison in the capture efficiency of L3.6pl cells between antibody-functionalized LFAM2 and antibody-functionalized LFAM. The difference between two devices is statistically not significant (NS) at the 95% confidence level at a low flow rate (0.5, 1.0, and 1.5 μL/s). However, LFAM generally shows higher capture efficiency than LFAM2 due to its smaller filter sizes and the difference between two devices is statistically significant (P<0.05) at a high flow rate (2.0 μL/s).

Size of CTCs captured in LFAM2

The size of CTCs captured in the LFAM2 device was measured using CellSens (Olympus, PA). Since CTCs are not exactly round, the maximum and minimum dimension of a CTC are measured. **Figure S5** shows the images of a representative CTC and a white blood cell (WBC) in LFAM2 while **Figure S6** shows the size distribution of CTCs, with an average of 14.8 μm for the maximum dimension and 10.9 μm for the minimum dimension.

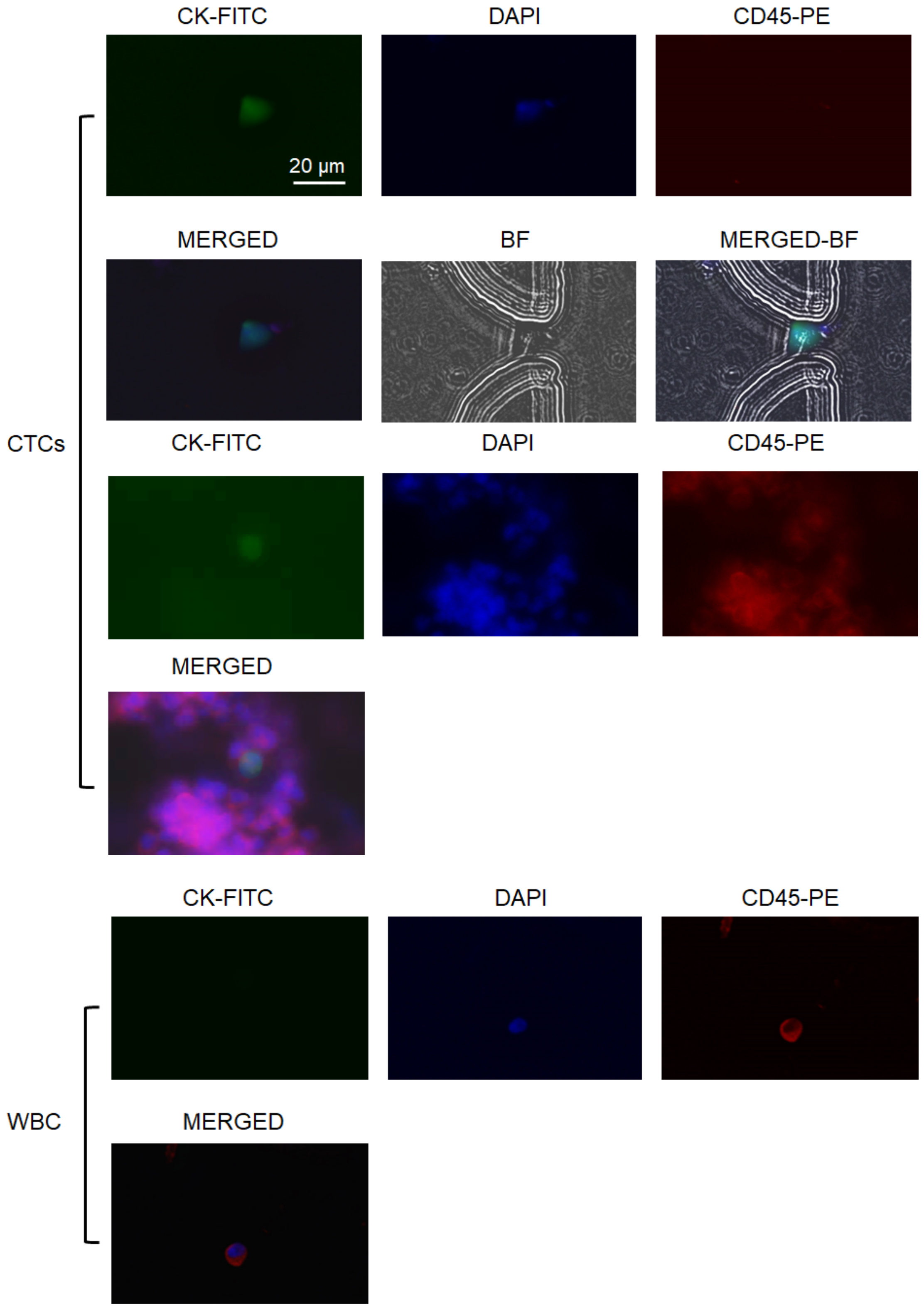

Figure S5. Representative images of CTCs and white blood cells in LFAM2. (Top) CTC (CK^+/$DAPI^+$/$CD45^-$); (Bottom) WBC (CK^-/$DAPI^+$/$CD45^+$).

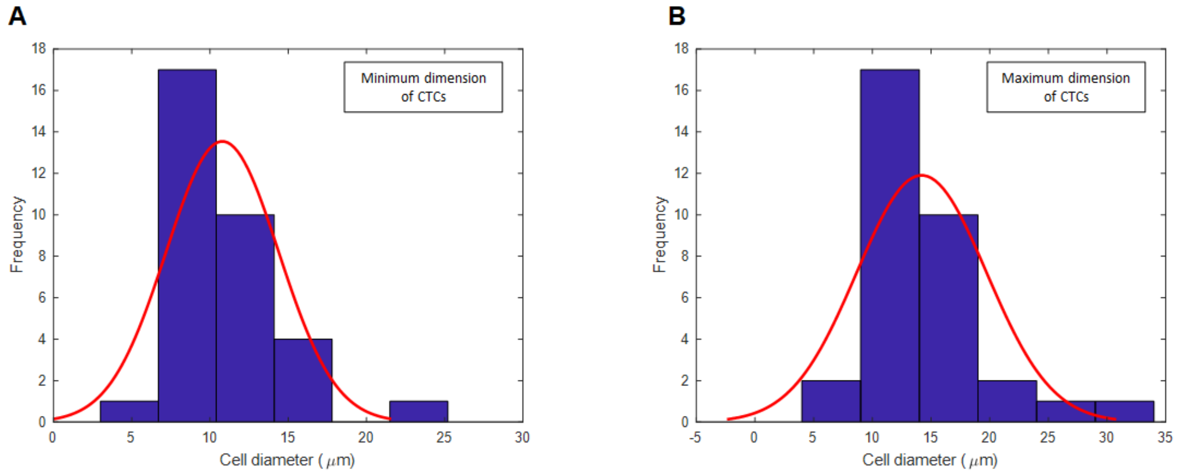

Figure S6. (A) The distribution of minimum dimensions of CTCs measured by CellSens; (B) The distribution of maximum dimensions of CTCs measured by CellSens.

References

1 Nagrath, S. *et al.* Isolation of rare circulating tumour cells in cancer patients by microchip technology. *Nature* **450**, 1235-1239 (2007).

2 Sheng, W. *et al.* Capture, release and culture of circulating tumor cells from pancreatic cancer patients using an enhanced mixing chip. *Lab on a chip* **14**, 89-98 (2014).

3 Stott, S. L. *et al.* Isolation of circulating tumor cells using a microvortex-generating herringbone-chip. *Proceedings of the National Academy of Sciences* **107**, 18392-18397, doi:10.1073/pnas.1012539107 (2010).

4 Wang, S. *et al.* Three-Dimensional Nanostructured Substrates toward Efficient Capture of Circulating Tumor Cells. *Angewandte Chemie* **121**, 9132-9135 (2009).

5 Zhang, N. *et al.* Electrospun TiO2 nanofiber-based cell capture assay for detecting circulating tumor cells from colorectal and gastric cancer patients. *Advanced Materials* **24**, 2756-2760 (2012).

6 Chen, K. *et al.* Integration of Lateral Filter Arrays with Immunoaffinity for Circulating-Tumor-Cell Isolation. *Angew Chem Int Ed Engl* **58**, 7606-7610, doi:10.1002/anie.201901412 (2019).
